# Guilt by association - what is the true risk of malignancy in children treated with etanercept for JIA?

**DOI:** 10.1186/1546-0096-8-23

**Published:** 2010-08-16

**Authors:** Randy Q Cron, Timothy Beukelman

**Affiliations:** 1 Department of Pediatrics, Division of Rheumatology, University of Alabama at Birmingham, Birmingham, AL 35294, USA

## Abstract

Recently, the Food and Drug Administration placed a "black box" label on etanercept, and other tumor necrosis factor inhibitors used to treat childhood arthritis, warning of the risk of malignancies. The Food and Drug Administration made their decision based on a review of 48 cases of malignancies identified worldwide in children treated with tumor necrosis factor inhibitors for inflammatory bowel disease, sarcoidosis, and juvenile idiopathic arthritis. Recently, an article in Pediatric Rheumatology demonstrated that there may not be an increased risk of cancer in children with juvenile idiopathic arthritis treated specifically with the tumor necrosis factor receptor fusion protein, etanercept. There are many confounding issues regarding whether or not etanercept increases the risk of malignancy, specifically lymphomas, above the background rate of cancer in children with juvenile idiopathic arthritis who are not being treated with biologic agents. Whether or not it was appropriate for the Food and Drug Administration to lump cancer patients with underlying granulomatous diseases (inflammatory bowel disease and sarcoidosis) with children with juvenile idiopathic arthritis is explored herein. Moreover, the amalgamation of etanercept with anti-tumor necrosis factor monoclonal antibodies (adalimumab and infliximab) is another point of contention. What is clear is that there is much that is currently unknown to be able to convincingly demonstrate a substantial risk of cancer in children with juvenile idiopathic arthritis treated with etanercept. Conversely, there is ample evidence demonstrating remarkable benefit of etanercept in treating juvenile idiopathic arthritis. Physicians treating childhood arthritis should weigh these potential risks and benefits with patients and their families discussing the current limitations in available data regarding the risk of cancer in children treated with etanercept for juvenile idiopathic arthritis.

## Introduction

The bench to bedside transition of tumor necrosis factor (TNF) inhibitors has been a truly remarkable breakthrough in the treatment of both adult and pediatric chronic inflammatory arthritis, and no less worthy than awarding the 2003 Lasker Clinical Medical Research Award to Drs. Feldman and Maini [1]. The first of these wonder drugs to be approved by the Food and Drug Administration (FDA) and used extensively to treat juvenile idiopathic arthritis (JIA) was the TNF receptor2-imumoglobulin Fc tail fusion protein, etanercept [2]. In clinical trials, etanercept has been shown to be safe and highly efficacious in treating JIA [3]. However, in November 2009, the FDA placed a new black box warning on TNF inhibitors, including etanercept, warning of the risks of malignancy. This was the result of the FDA identifying 48 cases of malignancy occurring in children associated with the use of the TNF inhibitors, infliximab (31 cases), adalimumab (2 cases), and etanercept (15 cases) [4]. The authors of this report estimated that the rate of overall malignancy associated with etanercept was approximately equal to the background rate in the general population, but the rate of lymphoma was approximately 5 times the background rate. Recently, employees of Amgen (makers of etanercept) and colleagues reported in this journal that there does not appear to be an overall increased risk of malignancy associated with etanercept but there may be an increased risk for lymphoma [5].

## Discussion

McCroskery and colleagues identified 18 malignancies worldwide among children who received etanercept, and 3 of these remain unconfirmed [5]. The potential risk window for a possible causal link between etanercept and cancer is unknown, but 5 cases had only received etanercept for 6 months or less prior to their cancer diagnoses. An even bigger question remains as to what proportion of the risk of cancer in patients with JIA is attributable to treatment with etanercept. Prior or concurrent treatment with certain non-biologic medications, such as methotrexate (which was received by 13 of the cases), may carry their own risks of lymphoma development in children [6] and adults [7] with chronic inflammatory arthritis.

Very recent independent preliminary studies have suggested that biologic naïve JIA patients experience an approximately 2- to 3-fold increased risk of cancer [8,9], and a nearly 4-fold increased risk of lymphoproliferative cancers [9]. This is similar to adults with rheumatoid arthritis where the disease itself confers a risk of lymphoma development [10]. Some recent large population studies have shown no additional risk of lymphoma conferred by TNF inhibitors in RA patients [11,12]. Thus, it is critical to know the background rate of malignancy in children with JIA; without this data, there is no way of deciphering either a protective effect or increased risk of lymphoma development in children with JIA treated with TNF inhibitors, such as etanercept.

What does seem likely, however, is a risk of developing hepatosplenic T cell lymphomas in children with inflammatory bowel disease (IBD) treated with a combination of azathioprine or 6-mercaptopurine (6-MP), and a monoclonal antibody (mAb) directed against TNF (e.g., infliximab or adalimumab) [13]. Although some children with IBD develop arthritis and are managed or co-managed by pediatric rheumatologists, it is more likely that pediatric rheumatologists will use methotrexate in combination with an anti-TNF mAb. This is relevant since all the lymphoma cases identified by the FDA in children with IBD on an anti-TNF mAb were also treated with the thiopurines, 6-MP or azathioprine [14]. Unfortunately, these particular lymphomas are often resistant to chemotherapy and are, therefore, frequently fatal [15]. Since granulomatous diseases (e.g., IBD, sarcoidosis) tend to respond better to anti-TNF mAb therapy [16], etanercept is rarely used to treat IBD. It remains unclear whether or not etanercept also contributes to increased risk of lymphomas in autoimmune disorders. Nevertheless, most all JIA patients treated with etanercept survived their various malignancies [4].

## Conclusions

It is often difficult for the FDA to choose when to alert the public about a potential drug-related safety issue as they rarely have adequate information to make definitive conclusions at the time of the warning. However, it remains questionable whether or not the FDA should have lumped different classes of TNF inhibitors and different patient populations with differing malignancies together and placed a generic black box warning on the TNF inhibitors for malignancy risk, particularly in the setting of JIA patients treated with etanercept. For rare diseases and rare events, it is extremely difficult to determine causality. Accordingly, interested parties have called for the creation of a consolidated registry to monitor the long-term safety of pediatric rheumatology treatments [17]. Until then, physicians and JIA patient families need to be aware of the current evidence, as well as the large gaps in data, to make intelligent informed decisions regarding weighing the clear benefits afforded by TNF inhibitors versus the potential risks of malignancy in children with JIA receiving etanercept therapy.

## List of abbreviations

Abbreviations are defined in the text where first used.

## Competing interests

The authors declare that they have no competing interests.

## Authors' contributions

RQC researched the subject and drafted the manuscript.

TB researched the subject and modified the manuscript accordingly.

Both authors read and approved the final manuscript.
